# Single-Lens Reconstructed Three-Dimensional Versus Two-Dimensional Visualization in Uniportal Full-Endoscopic Unilateral Laminotomy with Bilateral Decompression for Lumbar Spinal Stenosis: A Retrospective Comparative Study

**DOI:** 10.3390/life16101608

**Published:** 2026-09-25

**Authors:** Yu-Hsuan Chung, Hao-Chun Chuang, Ming-Hsien Hu, Hong-Lin Su

**Affiliations:** 1 Doctoral Program in Translational Medicine, National Chung Hsing University, Taichung 402, Taiwan; 2 Rong Hsing Translational Medicine Research Center, National Chung Hsing University, Taichung 402, Taiwan; 3 Department of Orthopedics, Show Chwan Memorial Hospital, Changhua 500, Taiwan; 4 Department of Life Sciences, National Chung Hsing University, Taichung 402, Taiwan; 5 Tainan Municipal Hospital (Managed by Show Chwan Medical Care Corporation), Tainan 701, Taiwan; 6 Department of Orthopedic Surgery, National Cheng Kung University Hospital, College of Medicine, National Cheng Kung University, Tainan 701, Taiwan; 7 Department of Post-Baccalaureate Medicine, College of Medicine, National Chung Hsing University, Taichung 402, Taiwan; 8 The iEGG and Animal Biotechnology Research Center, National Chung Hsing University, Taichung 402, Taiwan

**Keywords:** 3D reconstructed visualization, stereopsis, depth perception, neurogenic claudication, interlaminar approach, minimally invasive spine surgery, operative time, surgical training, patient-reported outcome measures

## Abstract

Background: Full-endoscopic unilateral laminotomy with bilateral decompression (ULBD) through a single portal lacks stereopsis, which dual-lens three-dimensional (3D) endoscopes cannot supply. This study evaluated whether a single-lens 3D reconstructed system shortens key operative steps, whether the benefit is greater with segmental deformity, and whether 12-month outcomes differ. Methods: Sixty-seven consecutive patients undergoing single-level uniportal full-endoscopic ULBD (2023–2024) were retrospectively reviewed; the same endoscope was used in two-dimensional (2D) mode (*n* = 33) or single-lens 3D mode (*n* = 34). Allocation was not randomized; assignment to the 2D or 3D group was determined by insurance coverage rather than by the investigators. Operative times were analyzed in all 67 patients; pain and function were analyzed in the 62 who completed 12-month follow-up. Deformity was present in 14 and 17 patients (*p* = 0.534). Times, pain, and function over 12 months were compared by two-way ANOVA with a system × deformity interaction. Results: 3D shortened time to drilling (4.4 vs. 6.1 min) and flavectomy (41.8 vs. 61.4 min; *p* < 0.001). The interval from initiation of drilling to completion of flavectomy—the deep portion of the procedure—was 17.9 min shorter with 3D (37.4 vs. 55.3 min; *p* < 0.001). The benefit depended on deformity (interaction *p* = 0.038): deformity prolonged that interval by 7.7 min with 2D but by 0.02 min with 3D. No complications or reoperations occurred. No statistically significant between-group difference in pain relief was detected, and the 12-month Oswestry Disability Index advantage with 3D (6.3 percentage points) fell below the minimum clinically important difference. Conclusions: Single-lens 3D reconstructed visualization shortens the deeper, working-channel portion of uniportal full-endoscopic ULBD and offsets the added time cost of deformed anatomy, but was not associated with a detectable improvement in clinical outcomes in this cohort.

## 1. Introduction

Decompression remains the mainstay of treatment for lumbar spinal stenosis once non-operative care fails [1]. Unilateral laminotomy with bilateral decompression (ULBD) decompresses both sides through a single corridor while preserving the contralateral musculature and most of the posterior tension band [2,3], and has migrated from open to tubular microscopic and then full-endoscopic execution, with randomized and pooled analyses showing non-inferior functional outcome and less blood loss [3,4,5,6].

Performing ULBD with uniportal full-endoscopic surgery, though efficacious, is technically demanding and difficult to master because of the absence of stereopsis, and a substantial period of training and case experience is required before proficiency is achieved [7,8,9,10]. When segmental deformity, including spondylolisthesis, scoliosis, or axial rotation, is present, the bony landmarks that orient the laminotomy are displaced from their expected positions, and endoscopic anatomy can become unrecognizable to the point that misinterpretation during laminotomy has produced iatrogenic fracture even in experienced hands [11]. Stereoscopic vision selectively reduces depth-related drilling error while leaving lateral accuracy unchanged [12], and binocular cues improve both the magnitude and the consistency of perceived depth even in scenes already rich in pictorial cues [13]. Increasing image resolution does not substitute for this, as 4K ultra-high-definition endoscopy improves image detail but does not generate binocular disparity [12].

In otolaryngology and laparoscopy, three-dimensional (3D) stereoscopic endoscopy improved depth perception and reduced simulated error rates with safety equivalent to two-dimensional (2D) endoscopy, and allowed novices to reach proficiency more quickly [13,14]. Translation to spine endoscopy is limited by hardware: conventional 3D endoscopes need two optical channels, and the resulting scope diameter cannot be accommodated in a uniportal construct, where the optic and the working channel share one portal. Stereoscopic spine endoscopy has consequently been reported only in biportal configurations, and only descriptively [15,16].

A single-lens 3D reconstructed endoscopic system was developed to remove this constraint, generating a stereoscopic image by shape-from-shading without increasing scope diameter or compromising working-channel calibre [17]; its application to uniportal lumbar surgery has so far been limited to a two-patient descriptive report, in which field of view and exposure were unchanged relative to the 2D view [7]. Because both display modes share the same optic, cannula, instruments, field of view, and exposure, a comparison between them isolates the contribution of stereoscopic visualization. A 3D cohort undergoing uniportal full-endoscopic ULBD was therefore compared with a 2D cohort. The primary objective of the current study is to determine whether 3D reconstructed endoscopy shortens operative time and improves procedural efficiency. Secondary objectives include whether it affects the trajectories of postoperative pain and functional recovery, and whether these effects are modified by the presence of segmental deformity at the operated level.

## 2. Materials and Methods

### 2.1. Study Design and Patients

This retrospective cohort study was conducted, analyzed, and reported in accordance with the STROBE (Strengthening the Reporting of Observational Studies in Epidemiology) statement for observational studies (Table S1). This study was approved by the Institutional Review Board of Show Chwan Memorial Hospital on 9 August 2023 (SCMH_IRB No. 1120707). Patients treated before the date of approval were included under an approved retrospective review of existing de-identified clinical records, for which the requirement for written informed consent was waived; all patients enrolled thereafter provided written informed consent using the approved consent form. Sixty-seven consecutive patients underwent single-level uniportal full-endoscopic ULBD for lumbar degenerative disease between 1 January 2023 and 31 December 2024. Five patients did not complete 12 months of follow-up and were excluded from the final analysis, leaving 62 patients for evaluation. Only single-level procedures were included so that the two visualization systems could be compared under equivalent operative conditions. Allocation to the three-dimensional or two-dimensional group was not randomized. Uniportal full-endoscopic unilateral laminotomy with bilateral decompression is not reimbursed by the Taiwanese National Health Insurance program, and all patients therefore self-funded the procedure at a cost of approximately NT$200,000; use of the single-lens 3D reconstructed system carried an additional charge of approximately NT$20,000. Patients whose private insurance coverage met this incremental cost were treated with the 3D reconstructed system, and all others underwent the two-dimensional procedure. All operations were performed by the same spine surgeon using an identical uniportal technique, and no patient-related, radiographic or surgeon-preference criteria were used to select the visualization modality. Because cases of both types occurred throughout the entire study period rather than in separate consecutive blocks, any effect of the surgeon’s evolving experience was distributed across the two groups. No a priori sample size calculation was performed, and all consecutive eligible patients treated during the study period were included. Inclusion criteria were: (1) persistent low back pain and radiating pain or claudication in the lower extremities despite 3 months of conservative treatment; (2) radiographic evidence of lumbar spinal stenosis (central or foraminal) corresponding to Schizas grade C or D on the axial view of T2 weighted lumbar MRI images; and (3) availability of complete preoperative and postoperative imaging and clinical data. Exclusion criteria were: (1) a history of spine surgery at adjacent levels; (2) radiographic evidence of segmental instability; (3) spondylolisthesis of Meyerding grade 2 or above; (4) prior spine infection; and (5) malignant or metastatic lesions of the spine.

### 2.2. Preoperative Evaluation

The level to be decompressed was determined by correlating the clinical symptoms with the imaging findings. All patients underwent plain radiography of the lumbar spine, including anteroposterior and dynamic flexion–extension lateral views, to assess segmental instability and disc height, and magnetic resonance imaging, on which the severity of central stenosis was graded according to the Schizas classification [18]. Preoperative radiographs were reviewed for spinal deformity at the operated level, which was defined as any of the following: (1) spondylolisthesis with up to 25% translation on the lateral view (Meyerding grade 1) [19]; (2) scoliosis, defined as a Cobb angle greater than 10 degrees on the anteroposterior view; or (3) rotation, defined as partial disappearance of the pedicle on the anteroposterior view (Nash–Moe grade 2 or above).

### 2.3. Visualization Systems

The two groups were defined by the visualization system used: (1) the 2D group, consisting of patients operated on using a conventional two-dimensional endoscopic camera and light source system (Camsource^®^ Duo camera and light source system; Joimax GmbH, Karlsruhe, Germany), and (2) the 3D group, consisting of patients operated on using the conventional endoscope augmented with a 3D reconstructed endoscopic image-analyzing system that generates a stereoscopic image pair from a single optical channel by real-time image analysis (Darwin MonoStereo^®^ MS-301 3D Endoscopic System, MedicalTek Co., Ltd., Taichung, Taiwan; hereafter, single-lens 3D reconstructed).

The single-lens 3D reconstructed system applies real-time 2D-to-3D conversion based on a shape-from-shading algorithm that estimates depth information directly from the light distribution and shading patterns within a standard 2D endoscopic image, without requiring a dual-lens or stereo-camera endoscope [20]. Depth is therefore recovered from a single optical image; no second optical channel, no second camera, and no tip-mounted dual video chip is involved. For each video frame, the direction of the illumination source is estimated and specular highlights are suppressed. Variations in pixel intensity are then used to compute a depth map of the surgical field, which is refined to preserve anatomical edges and limit distortion. From this depth map the system renders a paired left- and right-eye image and displays it on a compatible 3D monitor. Conventional monoscopic endoscopes are thereby converted into a stereoscopic visualization system in real time, without hardware modification of the endoscope. In practice, the surgeon gains a reconstructed impression of depth of the dural sac, ligamentum flavum, and bony margins of the laminotomy, using the same endoscope, working channel, and instruments as in conventional 2D surgery. Stereoscopic output was shown on a dedicated 3D monitor viewed through passive polarization glasses, positioned alongside the conventional 2D monitor (Figure 1). The system employs a single-lens (monocular) endoscope; the stereoscopic impression is generated by software-based 3D reconstruction rather than by two independent optical channels. It should therefore be understood as a 3D reconstructed, rather than a true stereoscopic (binocular) 3D, system: no binocular disparity is acquired optically, and the depth cues presented to the surgeon are computed from a single optical image rather than captured from two separate viewpoints.

### 2.4. Surgical Technique

All procedures were performed at a single tertiary medical center by one spine surgeon experienced in full-endoscopic techniques, whose annual volume was 100 to 150 endoscopic spine procedures. All operations were performed under general anesthesia, with the patient prone on a radiolucent operating table over a Wilson frame so that the lumbar spine was held in slight flexion. Before surgery, every patient received a detailed explanation of the operative technique, alternatives, expected benefits, anesthetic options, and postoperative rehabilitation program, and operative consent was obtained.

ULBD was performed through an outside-in, also commonly known as over-the-top uniportal approach [9]. Ipsilateral laminar drilling was carried out first to expose the deep layer of the ligamentum flavum while preserving the medial facet. After the superficial layer had been removed, the deep ligamentum flavum was deliberately left intact throughout the bony decompression so that it shielded the underlying neural elements. Midline access was then established by decompressing the base of the spinous process and identifying the “V” point of the cephalad lamina. Over-the-top translaminar decompression followed, with drilling of the ventral aspect of the spinous process, the contralateral caudal lamina, and the medial part of the superior articular facet. Once all superior, inferior, and lateral bony attachments had been released, the deep ligamentum flavum was resected en bloc, exposing the decompressed neural structures. Decompression was considered adequate when the traversing and exiting nerve roots were seen to pulsate freely on both the ipsilateral and the contralateral side, both pedicles had been visualized and palpated, and the lateral recess was confirmed to be widely open with the lateral edge of the traversing dura clearly visible bilaterally.

### 2.5. Outcome Measures

Patient data, including demographic information (age, sex, height, and weight) and operative parameters (operative level, laterality, hospital stay, and intraoperative timing details), were obtained from the patients’ electronic medical records.

The primary outcomes were two intraoperative time intervals, both measured from the timestamps of the archived endoscopic video recording of each procedure. Time to drilling was defined as the interval between skin incision and the start of laminotomy, and therefore reflects the time required to recognize the anatomical landmarks, achieve hemostasis, and adequately remove the overlying soft tissue. Time to flavectomy was defined as the interval between skin incision and complete removal of the ligamentum flavum, and therefore represents the total duration of the decompression. Both variables are therefore cumulative operative intervals measured from skin incision, and neither represents the duration of drilling or of flavectomy as a discrete surgical step. In addition, the interval from initiation of drilling to completion of flavectomy was derived as time to flavectomy minus time to drilling. This interval isolates the deep, working-channel portion of the procedure, but it remains a composite of several consecutive deep steps and is not a measure of flavectomy duration alone.

Secondary outcomes were patient-reported pain and disability, assessed with the Visual Analog Scale (VAS) for back and leg pain [21] and the Oswestry Disability Index (ODI) [22]. Both instruments were administered in the outpatient clinic as self-completed paper questionnaires, collected on the day before surgery and at 1, 3, 6, and 12 months postoperatively within a window of ±2 weeks for each time point. Questionnaires were distributed and checked for completeness by a research coordinator who was not involved in the surgical care and was blinded to the visualization modality. All patients attended every scheduled assessment, and no outcome data were missing. Intraoperative and postoperative complications and any reoperation at the index level within 12 months were also recorded.

### 2.6. Statistical Analysis

Descriptive statistics were used to summarize demographic and clinical data. Categorical variables were expressed as counts and proportions and compared using the chi-square test. Continuous variables were expressed as mean ± standard deviation (SD) and compared between groups using the Student *t*-test, with Welch’s correction applied when variances were unequal; variables with skewed distributions were compared using the Mann–Whitney U test. A two-sided *p*-value < 0.05 was considered statistically significant. Effect estimates are reported as mean differences with 95% confidence intervals. For time to drilling, Welch (unequal-variance) intervals with Welch–Satterthwaite degrees of freedom were used because the group variances were heterogeneous (F test *p* < 0.001); for the interval from initiation of drilling to completion of flavectomy, Student pooled-variance intervals were used, as equal variances were supported overall and within both deformity strata. Standardized effects are reported as Hedges’ g with the small-sample correction and its 95% confidence interval. Because intraoperative data were complete for all operated patients, the operative-time analyses (time to drilling, time to flavectomy, and the interval from initiation of drilling to completion of flavectomy) included all 67 patients, comprising the 5 patients who were subsequently lost to clinical follow-up; the ODI and VAS outcome analyses were restricted to the 62 patients with complete 12-month follow-up. All analyses were performed using Statistical Product and Service Solutions (SPSS) 26.0 (IBM Corp., Armonk, NY, USA).

To examine the effects of 3D reconstructed endoscopy use and the presence of spinal deformity on time to drilling and time to flavectomy, two-way analysis of variance (ANOVA) was performed, with endoscope type (2D vs. 3D) and deformity status (present vs. absent) as fixed factors and their interaction term included. Where a significant interaction was identified, simple main effects were examined to interpret the direction of the effect within each subgroup. Partial eta squared was reported as the effect size.

Serial changes in leg VAS, back VAS, and ODI across the five assessment points (preoperative and 1, 3, 6, and 12 months postoperatively) were analyzed using a mixed-design repeated-measures ANOVA, with time as the within-subject factor and endoscope type as the between-subject factor. The group × time interaction was treated as the primary test of whether recovery trajectories differed between groups; main effects of time and group were also reported. Sphericity was assessed, and corrected *p*-values are noted where correction affected the interpretation. Where the interaction was significant, post hoc between-group comparisons of change from baseline at each timepoint were performed with Bonferroni adjustment. In addition, analysis of covariance (ANCOVA) was used to compare 12-month leg VAS, back VAS, and ODI between groups, and adjusted means with 95% CIs are reported. The preoperative ODI served as the covariate in the ODI model. Because a single combined preoperative VAS was recorded rather than separate back and leg scores, that same value was used as the covariate in both the back VAS and the leg VAS models.

## 3. Results

### 3.1. Demographics

A total of 67 patients underwent surgery, 33 with the 2D system and 34 with the 3D system, and all 67 contributed to the operative-time analyses because intraoperative records were complete for every patient (Table 1). The clinical outcome analyses were restricted to the 62 patients who completed 12-month follow-up, 31 in each group (Table 2). Age, sex, anthropometric and hospital-stay comparisons could be computed only for the 62 patients with complete records. The groups differed in sex distribution (2D: 24 males and 7 females; 3D: 15 males and 16 females; *p* = 0.018) but did not differ significantly in age (63.6 ± 13.1 vs. 59.4 ± 17.3 years, *p* = 0.290), height (158.6 ± 8.3 vs. 161.4 ± 8.7 cm, *p* = 0.198), and weight (55.9 ± 11.0 vs. 60.4 ± 13.8 kg, *p* = 0.161). The most commonly operated level was L4/L5 (28/62), followed by L5/S1 (18/62), with similar distributions between groups (*p* = 0.637); the operated side was evenly distributed (left/right 16/15 in both groups, *p* = 1.000). Spinal deformity was present in 14/33 patients in the 2D group (42.4%) and 17/34 in the 3D group (50.0%; *p* = 0.534). Hospital stay (2.2 ± 0.7 vs. 2.3 ± 0.8 days, *p* = 0.600) and intraoperative blood loss, which could be recomputed in all 67 patients (58.0 ± 12.1 vs. 52.9 ± 14.1 mL, *p* = 0.12), did not differ significantly.

All three intraoperative intervals favored the 3D group. Time to drilling was shorter with the 3D system (6.1 ± 2.6 vs. 4.4 ± 1.2 min, *p* = 0.001), as was time to flavectomy (61.4 ± 8.0 vs. 41.8 ± 7.3 min, *p* < 0.001), representing reductions of 29% and 32%, respectively. The interval from initiation of drilling to completion of flavectomy was likewise shorter with the 3D system (55.3 ± 8.2 vs. 37.4 ± 7.2 min, *p* < 0.001), a 32% reduction. No dural tear or other intraoperative complication occurred in either group, and no patient required reoperation at the index level during the 12-month follow-up.

### 3.2. Effects of the 3D System and Spinal Deformity on Surgical Efficiency

For time to drilling, the effect of the 3D system did not depend on the presence of deformity (interaction F(1, 63) = 1.98, *p* = 0.165, partial η^2^ = 0.03). Time to drilling was significantly faster with the 3D system (6.1 vs. 4.4 min, a 28.6% reduction; F(1, 63) = 14.72, *p* < 0.001, partial η^2^ = 0.19), whereas deformity alone did not significantly prolong drilling (4.9 vs. 5.8 min; F(1, 63) = 3.46, *p* = 0.067, partial η^2^ = 0.05). Cell variances were heterogeneous for this outcome (largest-to-smallest ratio 5.81), so these F-test *p*-values should be regarded as approximate; a Welch *t*-test confirmed the endoscope effect (mean difference 1.76 min, 95% CI 0.7 to 2.8; t = 3.49, df = 44.3, *p* = 0.001; Hedges’ g 0.85, 95% CI 0.35 to 1.35). Time to drilling was also considerably less variable with the 3D system (SD 1.2–1.3 vs. 2.4–2.8 min), indicating more consistent performance.

For time to flavectomy, the effect of the 3D system did depend on deformity status (interaction F(1, 63) = 6.79, *p* = 0.011, partial η^2^ = 0.10). Deformity prolonged flavectomy by 9.3 min in the 2D group (57.5 vs. 66.8 min, *p* < 0.001) but by only 0.3 min in the 3D group (41.7 vs. 41.9 min, *p* = 0.92), indicating that the 3D system largely offset the additional difficulty of deformed anatomy. Flavectomy was markedly faster overall with the 3D system (61.4 vs. 41.8 min; F(1, 63) = 137.94, *p* < 0.001, partial η^2^ = 0.69), and deformity prolonged the procedure overall (50.0 vs. 53.2 min; F(1, 63) = 7.55, *p* = 0.008, partial η^2^ = 0.11), although this reflected the 2D group almost entirely.

The interval from initiation of drilling to completion of flavectomy, which isolates the deep, working-channel portion of the procedure, showed the same pattern. This interval was 17.9 min shorter with the 3D system (55.3 vs. 37.4 min, a 32.3% reduction; F(1, 63) = 104.75, *p* < 0.001, partial η^2^ = 0.62), and the effect again depended on deformity status (interaction F(1, 63) = 4.49, *p* = 0.038, partial η^2^ = 0.07). Deformity prolonged the interval by 7.7 min in the 2D group (52.0 vs. 59.7 min, *p* = 0.006) but by 0.02 min in the 3D group (37.4 vs. 37.4 min, *p* = 0.99), so the penalty associated with deformed anatomy was essentially abolished under 3D visualization. Deformity prolonged the interval overall (F(1, 63) = 4.55, *p* = 0.037, partial η^2^ = 0.07), again reflecting the 2D group alone, and the 3D advantage was significant both in the absence and in the presence of deformity (both *p* < 0.001). Cell variances were homogeneous for this outcome (ratio 1.33, F-test *p* = 0.43). Group means were 55.29 ± 8.23 min (95% CI 52.4 to 58.2) with 2D and 37.42 ± 7.17 min (95% CI 34.9 to 39.9) with 3D, a mean difference of 17.87 min (95% CI 14.1 to 21.6; t(65) = 9.49, *p* < 0.001; Hedges’ g 2.29, 95% CI 1.67 to 2.91). Stratified by deformity, the 3D advantage was 14.63 min in its absence (95% CI 9.4 to 19.8, a 28.1% reduction; Hedges’ g 1.87, 95% CI 1.08 to 2.66) and 22.28 min in its presence (95% CI 17.2 to 27.4, a 37.3% reduction; Hedges’ g 3.12, 95% CI 2.05 to 4.20). The two stratum intervals overlap, so the interaction reflects a difference in the magnitude of the 3D advantage rather than in its direction—3D was faster in both strata.

### 3.3. Effects of the 3D System on Postoperative Pain Relief

Both leg and back pain improved markedly over 12 months (leg VAS: F(4, 240) = 135.26, *p* < 0.001; back VAS: F(4, 240) = 152.32, *p* < 0.001; Figure 2). Neither outcome showed a between-group main effect (leg VAS F(1, 60) = 1.59, *p* = 0.213; back VAS F(1, 60) = 0.39, *p* = 0.536), and recovery trajectories did not differ between groups for either outcome (leg VAS group × time F(4, 240) = 1.18, *p* = 0.320; back VAS F(4, 240) = 1.52, *p* = 0.197). At 12 months, neither back pain (2.3 vs. 2.7, adjusted difference 0.4, 95% CI −0.1 to 0.9; *p* = 0.150; Figure 2A) nor leg pain (2.4 vs. 2.0, adjusted difference −0.4, 95% CI −0.9 to 0.2; *p* = 0.177; Figure 2B) differed significantly between the 3D and 2D groups. Pain improved substantially in both groups, and no statistically significant between-group difference was detected at one year.

### 3.4. Effects of the 3D System on Functional Recovery

ODI improved from 60.4% preoperatively to 20.9% at 12 months (F(4, 240) = 185.80, *p* < 0.001; Figure 3). Overall ODI did not differ between groups (*p* = 0.630), but recovery trajectories diverged (group × time F(4, 240) = 2.93, *p* = 0.022), with the difference becoming most apparent at later follow-up. At 12 months, ODI was significantly lower in the 3D group (17.7% vs. 24.0%; adjusted difference 6.3 percentage points, 95% CI 3.0–9.5; F(1, 59) = 14.77, *p* < 0.001, partial η^2^ = 0.20). Thus, while no statistically significant between-group difference in pain outcomes was detected, the 3D system was associated with a modest functional advantage of approximately 6 ODI percentage points at one year, the clinical relevance of which requires confirmation in a larger cohort.

## 4. Discussion

Endoscopic lumbar decompression is an increasingly popular treatment for lumbar spinal stenosis. In the present study, full-endoscopic ULBD performed with a single-lens 3D reconstructed uniportal endoscopic system was compared against the same endoscope operated in 2D mode. Three findings emerged. First, the 3D system shortened both intraoperative intervals measured, most markedly for flavectomy and in patients with segmental deformity. Second, no statistically significant between-group difference in pain relief was detected. Third, no overall between-group difference in functional recovery was detected, although the ODI trajectories diverged in favor of the 3D group. No intraoperative complication occurred in either group. Clinically, the 3D reconstructed system offers a reproducible reduction in the time required to complete flavectomy and bony decompression, at no apparent cost to safety or to one-year pain relief. Its effect on functional outcome remains exploratory and requires prospective confirmation, but the gain requires no change in portal strategy or instrumentation and is readily absorbed into existing workflows.

In the present series the single-lens 3D reconstructed system shortened every intraoperative interval measured. Time to drilling fell from 6.1 ± 2.6 to 4.4 ± 1.2 min (*p* = 0.001) and time to flavectomy from 61.4 ± 8.0 to 41.8 ± 7.3 min (*p* < 0.001), while the interval from initiation of drilling to completion of flavectomy—the portion of the procedure conducted through the working channel—fell from 55.3 ± 8.2 to 37.4 ± 7.2 min, a mean saving of 17.9 min (95% CI 14.1 to 21.6; Hedges’ g 2.29; *p* < 0.001). The saving was largest where the anatomy was most distorted: 22.3 min (37.3%) in the presence of segmental deformity against 14.6 min (28.1%) in its absence, and the deformity-related time penalty evident under 2D visualization was essentially abolished under 3D. Acceleration of endoscopic work by a stereoscopic display has been reported in other specialties. Harada et al. found that task completion time and instrument path length among laparoscopic experts were shorter on a 3D display than on a 2D display of comparable or higher resolution, indicating that the benefit derives from binocular disparity rather than from image resolution alone [12]. Other trials extended the benefit to surgeons in the early phase of training, showing that a 3D system improved the laparoscopic technique of novices [23,24]. Further studies and a meta-analysis supported these observations and attributed the shorter operative time and greater efficiency of movement to improved depth perception rather than to a general novelty effect [25]. Similar conclusions have been reached in otorhinolaryngology, where 3D endoscopy shortened the time to complete a task and conveyed anatomical detail and depth relationships more clearly than 2D endoscopy [14,26]. The present findings are consistent with this literature in direction, and extend it by localizing the benefit: within a single procedure, the time saved was concentrated in the step that depends most heavily on depth judgement, and in the patients whose anatomy made that judgement hardest. No prior spinal series compared an objective operative endpoint between 3D and 2D; the operative-time advantages invoked in that literature came from thyroid and donor-nephrectomy surgery [15].

The pattern of the observed effect supports this interpretation. Uniportal endoscopic ULBD is performed through a single working channel in which the endoscope and the instruments share one axis, so the surgeon must infer the third dimension from motion parallax and from the known geometry of the instruments. Two features of the procedure make this inference costly. When segmental deformity is present, the interlaminar window is rotated and foreshortened relative to the expected orientation, and the bony landmarks used to confirm position are displaced from their canonical relationships. When spondylolisthesis is present, the target structures lie further from the working channel, so a given angular error at the instrument tip translates into a larger positional error at the tissue. A stereoscopic display restores binocular depth cues and improves spatial awareness, allowing anatomical relationships to be read directly rather than reconstructed and instruments to be navigated to a target more accurately. Consistently with this account, the effect of the 3D system on flavectomy was large and depended on deformity status, so the 3D system largely offset the additional difficulty of deformed anatomy. For drilling, a step guided as much by tactile and fluoroscopic cues as by stereopsis, the effect was smaller and did not depend on deformity. Drilling times were also considerably less variable with the 3D system, indicating that the initial phase of the procedure became more predictable as well as faster—a property of practical value for operative scheduling independent of the change in mean duration.

The clinical consequences of this efficiency gain, nevertheless, should not be overstated. No statistically significant between-group difference in pain relief was detected: neither leg nor back pain showed a between-group main effect, recovery trajectories did not diverge, and the 12-month differences were well within the confidence limits of no difference and far below the minimum clinically important difference (MCID) for pain scales in lumbar spine surgery [27]. Importantly, the absence of a detectable difference held irrespective of deformity status, indicating that patients with more complex anatomy were not disadvantaged by the 3D approach even though their operations were completed faster—the time saved was not purchased at the expense of decompression quality as reflected in symptom relief. Functional recovery requires more careful reading. Overall ODI did not differ between groups, and the 12-month advantage emerged from a significant group × time interaction rather than from a prespecified between-group comparison. This difference is also smaller than the accepted MCID for the ODI in this population [27], so it should be regarded as hypothesis-generating rather than as evidence of a clinically meaningful functional benefit. Attribution of this difference to more complete decompression is deliberately avoided, since no radiographic measure of decompression adequacy was obtained in this study; establishing such a link would require postoperative cross-sectional area measurements that the present retrospective dataset does not contain.

No dural tear, neural injury, or other intraoperative complication occurred in either group, and there were no reoperations at the index level during follow-up. Intraoperative blood loss was numerically lower in the 3D group but did not differ significantly, and hospital stay did not differ significantly. These observations are reassuring but should be interpreted with the precision the sample size allows: with 31 patients per group and no events observed, the upper bound of the 95% CI for the complication rate remains above 10%, so the data establish only that no safety signal was detected, not that the 3D system is safer or that rare complications occur at equal frequency. What can be stated is that the shorter operative intervals were achieved without any detectable increase in intraoperative injury, which is the relevant question when a new visualization platform is introduced into an established technique. For comparison, Heo et al. recorded no neural injury or durotomy in 38 patients, but two mild postoperative epidural haematomas and one transient hypesthesia [15].

The benefit observed here was obtained through a single working channel, using the standard uniportal instrument set and without any change in portal strategy—a departure from the way stereoscopic visualization has hitherto been delivered in endoscopic spine surgery. Stereoscopic endoscopy has conventionally depended on dual-channel optics: either the scope is connected to two video cameras, or two video chips are mounted at its tip, so that two images can be delivered to a stereoscopic display [17]. Heo et al. applied this approach to biportal lumbar decompressive laminectomy and found that it improved visualization during ligamentum flavectomy and during ipsilateral and contralateral nerve root decompression [15]. That series was single-arm: 38 patients, only 13 of them ULBD, studied with a dual-lens optical 3D endoscope, in which observers graded 3D vision poor before laminotomy but significantly superior after dura exposure; the present series required no such switching. No objective operative endpoint was compared between the two visualization modes. The comparison presented here is therefore complementary rather than duplicative: the same procedure was performed under 3D and under 2D visualization in two concurrent groups, and the comparison rests on timed intraoperative intervals rather than on observer preference. Because two spatially separated objectives require a lens 4 mm in diameter, those authors concluded that 3D optical technology was at that time applicable only to lenses of that size, and that 3D endoscopic spine surgery was therefore possible only with biportal approaches, in which the endoscope and the working instruments occupy separate portals [16]. The system used in the present study removes that constraint by deriving the stereoscopic image algorithmically from a single-lens optical train: the operative setup is that of standard 2D uniportal endoscopic surgery apart from the 3D monitor, its console, and circular polarized glasses. Feasibility of this approach in lumbar decompression was previously suggested by a single case report [7], and the present study extends that observation to a direct comparison with 2D visualization in a consecutive series. This is, however, a statement about hardware compatibility rather than about comparative effectiveness: the study included no biportal arm, so no inference about the relative merits of uniportal and biportal 3D decompression can be drawn from these data. What the results do show is that the visualization benefit previously described in a biportal 3D system is reproducible in a uniportal one and measurable against an objective operative endpoint, including within the deeper, working-channel portion of the procedure, which widens the range of endoscopic practice in which it can be adopted.

Surgeon-side tolerability of the stereoscopic display also merits comment. Stereoscopic visualization was used for the entire duration of every 3D case, and no procedure required reversion to the conventional 2D monitor. The operating surgeon nonetheless noted occasional eye strain and a transient sense of 3D-related dizziness, more noticeably during longer cases; these symptoms did not interrupt any procedure and resolved after surgery. Eye strain, dizziness, and a slightly darkened field were likewise reported from the dual-lens biportal series [15]. This is consistent with the visual fatigue described with stereoscopic displays in other surgical fields, and prospective measurement of surgeon visual fatigue would be a worthwhile component of future studies of single-lens 3D reconstructed endoscopy.

This study has several limitations. First, allocation was not randomized but determined by insurance coverage, which raises the question of selection bias. The scope for socioeconomic confounding is nonetheless narrow. Full-endoscopic ULBD is not reimbursed by the Taiwanese National Health Insurance program in any configuration, so every patient in both groups had already elected to self-fund an operation costing approximately NT$200,000; the 3D system added approximately NT$20,000, an incremental 10%. Both groups therefore cleared the same substantial financial threshold, and what separated them was willingness to meet a marginal surcharge rather than the ability to afford endoscopic surgery at all. Residual socioeconomic confounding can nevertheless not be excluded. Consistent with this, the groups did not differ in age, height, weight, operated level, operated side, or deformity prevalence. Unmeasured confounding cannot be excluded, and its likely direction would favor the 3D group, but its plausible magnitude is small. Second, the sex distribution differed significantly between the two groups. Third, no a priori sample size calculation was performed, as all consecutive eligible patients treated during the study period were included; the resulting cohort of 62 patients, 31 per group, was not powered to detect small between-group differences. The non-significant clinical-outcome comparisons should be interpreted as inconclusive rather than as demonstrating equivalence. The deformity comparison rested on 14 patients in the 2D group and 17 in the 3D group and is therefore exploratory and hypothesis-generating rather than definitive. Fourth, all procedures were performed by a single surgeon over a two-year period. Although 2D and 3D cases were distributed across the entire study period, a learning effect over time cannot be fully excluded. Fifth, only one combined preoperative VAS was recorded, so postoperative back and leg pain were each compared against a shared baseline, limiting the precision of site-specific change estimates. Sixth, follow-up was both short and incomplete. Twelve months is sufficient to capture recovery from decompression but not adjacent-segment degeneration, recurrent stenosis, or late instability, so a late harm attributable to either system would not be visible here; because a newer technique is more likely to be credited with a benefit than debited with a delayed complication, this censoring works in favor of the 3D system. In addition, 5 of 67 otherwise eligible patients (7.5%) did not reach the 12-month visit and were therefore excluded from the clinical outcome analyses, although their complete intraoperative records were retained in the operative-time analyses, and attrition of this kind is rarely random, since patients who recover completely and patients who fare poorly both attend less reliably than those in between. The analyzed cohort may therefore overstate the durability of both techniques, although with attrition below 10% and no difference in follow-up protocol between groups, the effect on the between-system comparison should be limited. Seventh, time to drilling and time to flavectomy are cumulative intervals measured from skin incision rather than the durations of the individual surgical steps. The interval from initiation of drilling to completion of flavectomy isolates the deeper portion of the procedure, but it is not the duration of flavectomy itself: unilateral flavectomy is frequently completed before the contralateral side is approached, requiring drilling to be resumed thereafter, so the interval still contains drilling time and would overestimate the time consumed by flavectomy alone. It remains a composite of several deep operative steps rather than a direct measure of stereoscopic depth perception. Eighth, surgeon-side tolerability of the stereoscopic display was not assessed systematically. Occasional eye strain and transient 3D-related dizziness were noted by the operating surgeon, but they were not recorded prospectively or measured with a validated instrument, so no quantitative statement about their frequency or severity is possible. Finally, no cost-effectiveness analysis was performed, and the unreimbursed charge for the 3D system must be weighed against the time saved during flavectomy and bony decompression. Operative efficiency was assessed from the timing of three defined operative intervals rather than from total operative time, which was not recorded in a form suitable for analysis; the effect of 3D visualization on the duration of the whole procedure therefore remains undetermined.

Future research should test 3D visualization in more complex procedures—multi-level decompression, revision surgery, and marked anatomical distortion. Its effect on the time and case volume required to master the technique, and on training, also warrants study, since in otolaryngology 3D endoscopy has already been reported to be superior to 2D endoscopy as a training tool [26].

## 5. Conclusions

In uniportal full-endoscopic ULBD, single-lens 3D visualization shortened the deeper, working-channel portion of the procedure, as well as the cumulative intervals to drilling and to flavectomy, without altering one-year pain relief and without any detectable safety cost. The practical implication is narrower than a general claim of superiority. Surgeons already proficient in 2D uniportal ULBD can expect the largest return where segmental deformity distorts the interlaminar anatomy, since that is where the time saved was concentrated; they should not, however, expect a patient-reported outcome benefit, because the functional advantage observed fell below the MCID and arose from an interaction analysis. Because the system attaches to a standard single-channel endoscope and uses the standard uniportal instrument set, adopting it does not require converting to a biportal platform. Two questions remain: whether the time saved during flavectomy and bony decompression justifies the unreimbursed cost of the technology, and whether the functional signal is real. Both call for a prospective randomized trial that collects cost data and separate baseline back and leg pain scores.

## Figures and Tables

**Figure 1:**
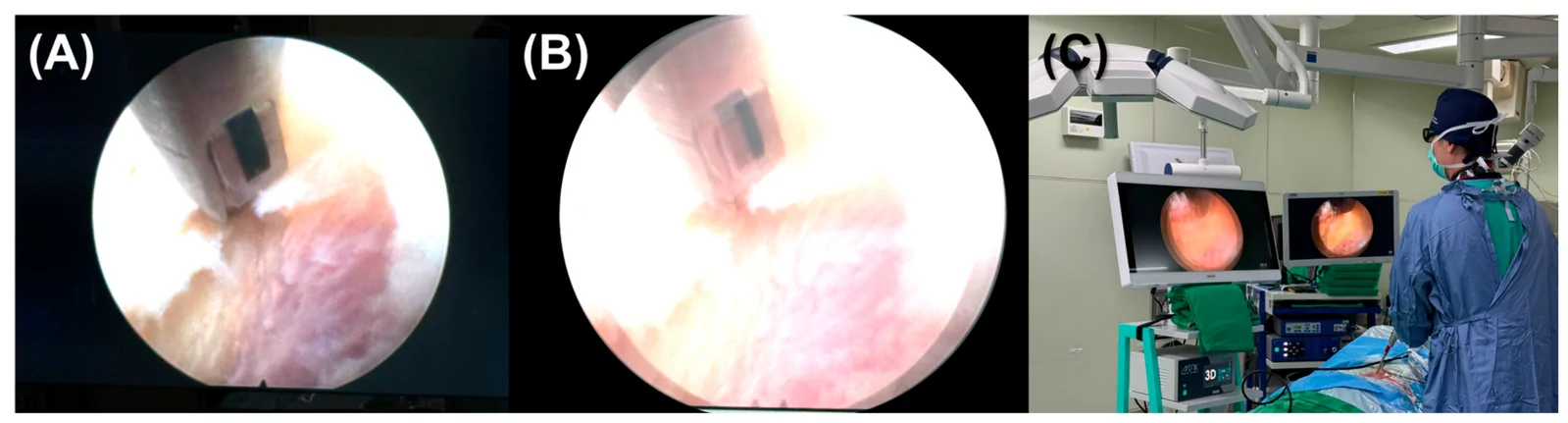
Intraoperative visualization during full-endoscopic unilateral laminotomy with bilateral decompression. (**A**) a conventional two-dimensional (2D) image and (**B**) the corresponding single-lens 3D reconstructed image. (**C**) Operating room setup: the 3D monitor on the left and the conventional 2D monitor on the right, both displaying the same endoscopic feed. The surgeon wears passive polarization glasses to view the stereoscopic image.

**Figure 2:**
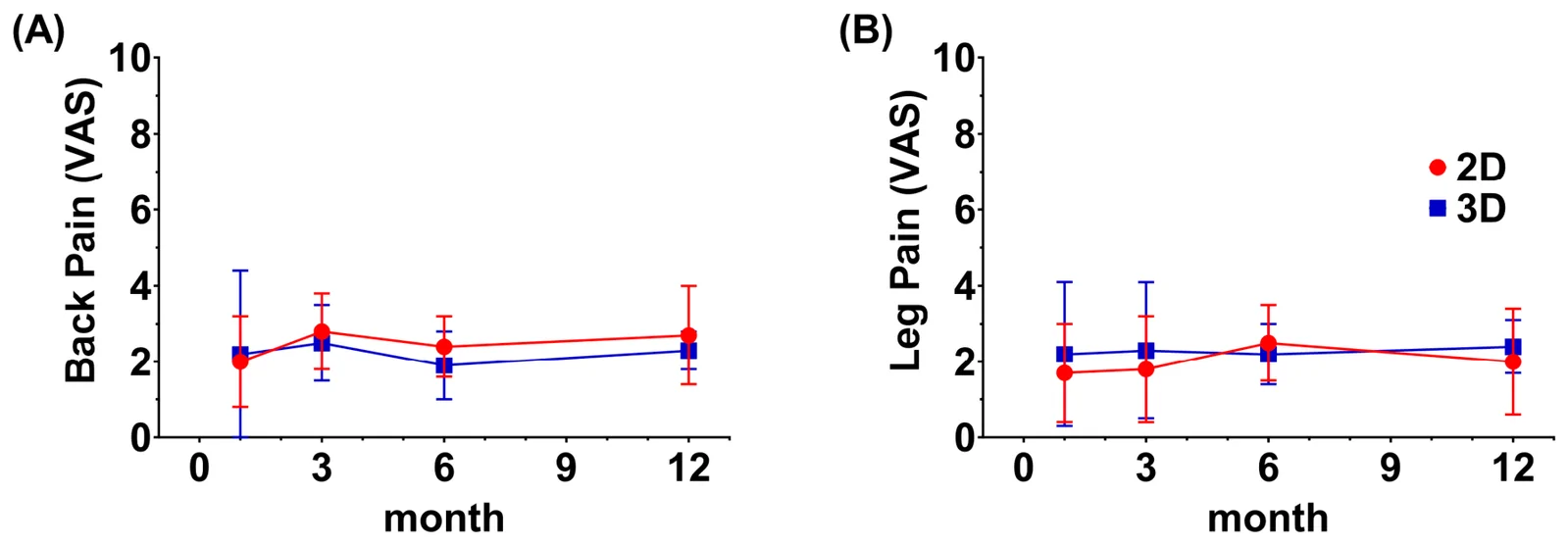
Back and leg pain over 12 months in the 2D and 3D groups. (**A**) Back pain and (**B**) leg pain on the 10-point visual analog scale (VAS), recorded at 1, 3, 6, and 12 months after surgery and analyzed as separate back- and leg-pain trajectories; the single combined preoperative VAS was used only as a covariate and is not plotted. Pain fell steeply in both groups (effect of time: back VAS F(4, 240) = 152.32; leg VAS F(4, 240) = 135.26; both *p* < 0.001), with no difference between groups (back *p* = 0.536; leg *p* = 0.213) and no difference in recovery trajectory (group × time: back *p* = 0.197; leg *p* = 0.320) on mixed-design repeated-measures ANOVA. Points are group means and error bars 1 SD (2D, red circles, *n* = 31; 3D, blue squares, *n* = 31).

**Figure 3:**
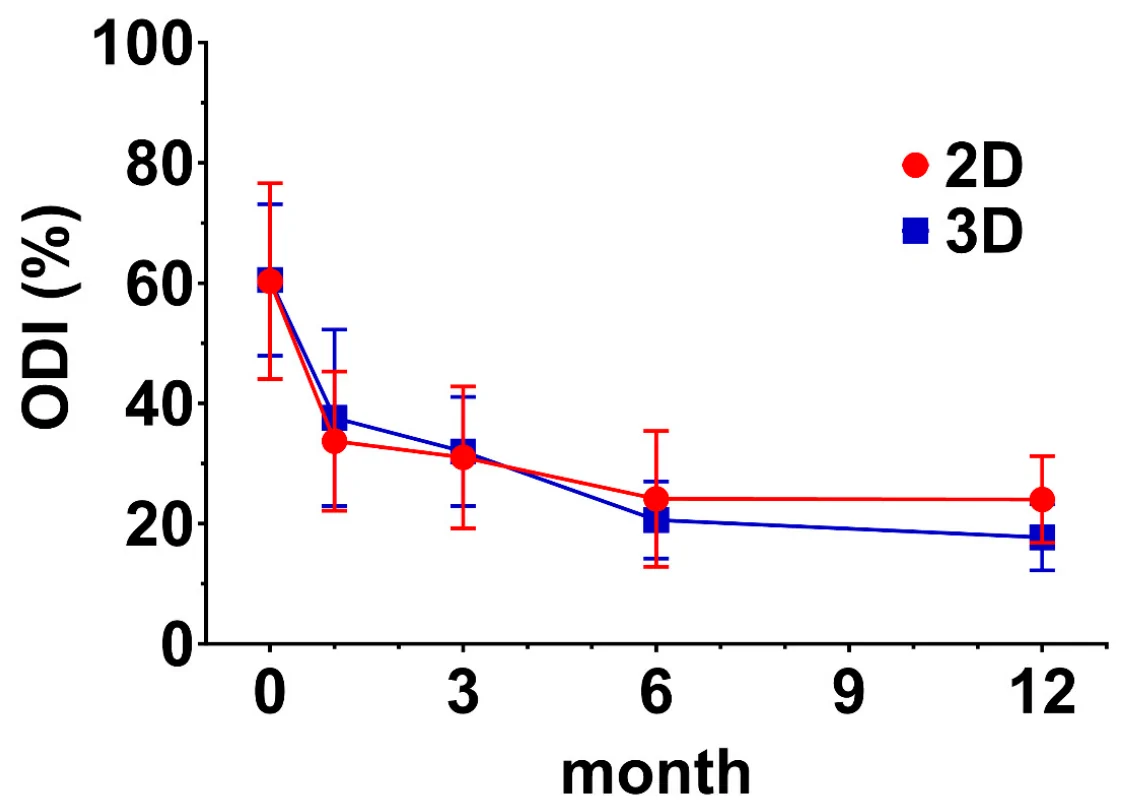
Oswestry Disability Index (ODI) over 12 months in the 2D and 3D groups. ODI was recorded preoperatively (month 0) and at 1, 3, 6, and 12 months after surgery. The recovery trajectories diverged (group × time F(4, 240) = 2.93, *p* = 0.022): the curves separate after 3 months, with continued improvement in the 3D group and a plateau in the 2D group. At 12 months ODI was lower in the 3D group (17.7% vs. 24.0%; adjusted difference 6.3 percentage points, 95% CI 3.0–9.5, *p* < 0.001). Points are group means and error bars 1 SD (2D, red circles, *n* = 31; 3D, blue squares, *n* = 31).

**Table 1:** Baseline demographic and perioperative characteristics of the two groups.

Variable	2D Group	3D Group	*p*-Value
Patients operated, *n*	33	34	—
Patients with 12-month follow-up, *n*	31	31	—
Age, years	63.6 ± 13.1	59.4 ± 17.3	0.290
Sex, male/female, *n*	24/7	15/16	0.018
Height, cm	158.6 ± 8.3	161.4 ± 8.7	0.198
Weight, kg	55.9 ± 11.0	60.4 ± 13.8	0.161
Operated side, left/right, *n*	16/15	16/15	1.000
Operated level distribution	—	—	0.637
Spinal deformity, *n* (%)	14/33 (42.4)	17/34 (50.0)	0.534
Hospital stay, days	2.2 ± 0.7	2.3 ± 0.8	0.600
Intraoperative blood loss, mL	58.0 ± 12.1	52.9 ± 14.1	0.12

Values are mean ± standard deviation unless stated otherwise. Age, sex, height, weight and hospital stay were computed in the 62 patients with complete records; deformity prevalence and blood loss in all 67 operated patients. The most frequently operated level was L4/L5 (28/62), followed by L5/S1 (18/62), with similar distributions between groups. Body mass index was not recorded; height and weight are reported separately.

**Table 2:** Comparison of clinical outcomes between the 2D and 3D reconstructed endoscopy groups (*n* = 62; the patients who completed 12-month follow-up).

	2D Group (Mean ± SD)	3D Group (Mean ± SD)	*p*-Value
Preoperative VAS (combined; covariate)	6.2 ± 1.7	6.5 ± 1.9	0.481
Preoperative ODI (%)	60.3 ± 16.3	60.5 ± 12.6	0.945
1-month Back VAS	2.0 ± 1.2	2.2 ± 2.2	0.386
1-month Leg VAS	1.7 ± 1.3	2.2 ± 1.9	0.381
1-month ODI (%)	33.7 ± 11.6	37.6 ± 14.7	0.410
3-month Back VAS	2.8 ± 1.0	2.5 ± 1.0	0.119
3-month Leg VAS	1.8 ± 1.4	2.3 ± 1.8	0.183
3-month ODI (%)	31.0 ± 11.8	32.0 ± 9.1	0.711
6-month Back VAS	2.4 ± 0.8	1.9 ± 0.9	0.050
6-month Leg VAS	2.5 ± 1.0	2.2 ± 0.8	0.157
6-month ODI (%)	24.1 ± 11.3	20.6 ± 6.4	0.136
12-month Back VAS	2.7 ± 1.3	2.3 ± 0.5	0.130
12-month Leg VAS	2.0 ± 1.4	2.4 ± 0.7	0.204
12-month ODI (%)	24.0 ± 7.2	17.7 ± 5.5	<0.001

VAS, Visual Analog Scale; ODI, Oswestry Disability Index. Values are mean ± standard deviation. A single combined preoperative VAS was recorded; separate preoperative back and leg scores were not available, so this value was used only as a covariate in the pain models and not as a baseline time point. Between-group comparisons used the Student *t*-test, except at 1 month for back VAS, leg VAS, and ODI, where the Mann–Whitney U test was used because of skewed distributions. The *p*-values shown are unadjusted; the pre-specified test of differing recovery trajectories was the group × time interaction of the repeated-measures ANOVA. After Holm correction for the 15 comparisons, only the difference in 12-month ODI remained significant (adjusted *p* = 0.004).

## Data Availability

The data presented in this study are available on reasonable request from the corresponding author. The data are not publicly available owing to institutional restrictions on the sharing of patient records.

## Supplementary Materials

The following supporting information can be downloaded at: https://www.mdpi.com/article/10.3390/life16101608/s1, Table S1: STROBE checklist.

## Abbreviations

The following abbreviations are used in this manuscript:

2D	Two-dimensional
3D	Three-dimensional
ANOVA	Analysis of variance
CI	Confidence interval
MCID	Minimum clinically important difference
ODI	Oswestry Disability Index
SD	Standard deviation
ULBD	Unilateral laminotomy with bilateral decompression
VAS	Visual Analog Scale
