# Supplementary material for: Single-Lens Reconstructed Three-Dimensional Versus Two-Dimensional Visualization in Uniportal Full-Endoscopic Unilateral Laminotomy with Bilateral Decompression for Lumbar Spinal Stenosis: A Retrospective Comparative Study
Source: Life (Basel). 2026 Sep 25;16(10):1608. doi: 10.3390/life16101608 (PMC13706338; doi:10.3390/life16101608)
Supplement: Supplementary file 1 [file life-16-01608-s001.zip › life-4534032-supplementary.pdf]

STROBE Statement—checklist of items that should be included in reports of observational studies

	Item No.	Recommendation	Page No.	Relevant text from manuscript
**Title and abstract**	1	(*a*) Indicate the study's design with a commonly used term in the title or the abstract	1	Single-Lens Reconstructed Three-Dimensional Versus Two-Dimensional Visualization in Uniportal Full-Endoscopic Unilateral Laminotomy with Bilateral Decompression for Lumbar Spinal Stenosis: A Retrospective Comparative Study
		(*b*) Provide in the abstract an informative and balanced summary of what was done and what was found	1	Single-lens 3D reconstructed visualization shortens the deeper, working-channel portion of uniportal full-endoscopic ULBD and offsets the added time cost of deformed anatomy, but should not be expected to improve clinical outcomes.
**Introduction**				
Background/rationale	2	Explain the scientific background and rationale for the investigation being reported	2	A single-lens 3D reconstructed endoscopic system was developed to remove this constraint, generating a stereoscopic image by shape-from-shading without increasing scope diameter or compromising working-channel calibre [17]; its application to

				uni-portal lumbar surgery has so far been limited to a two-patient descriptive report, in which field of view and exposure were unchanged relative to the 2D view [7]. Because both display modes share the same optic, cannula, instruments, field of view, and ex-posure, a comparison between them isolates the contribution of stereoscopic visualiza-tion.
Objectives	3	State specific objectives, including any prespecified hypotheses	2	A 3D cohort undergoing uniportal full-endoscopic ULBD was therefore compared with a 2D cohort to determine whether single-lens 3D reconstructed endoscopy improves procedural efficiency, whether it affects pain and functional recovery, and whether these effects are modified by the presence of segmental deformity at the operated level.
**Methods**				
Study design	4	Present key elements of study design early in the paper	3	Patients treated before the date of approval were included under an approved retro-spective review of existing de-identified clinical records, for which the requirement for written

				informed consent was waived; all patients enrolled thereafter provided written informed consent using the approved consent form
Setting	5	Describe the setting, locations, and relevant dates, including periods of recruitment, exposure, follow-up, and data collection	3	Sixty-seven consecutive patients underwent single-level uniportal full-endoscopic ULBD for lumbar degenerative disease between January 1, 2023, and December 31, 2024. Five patients did not complete 12 months of follow-up and were excluded from the final analysis, leaving 62 patients for evaluation. Only single-level procedures were included so that the two visualization systems could be compared under equivalent operative conditions.
Participants	6	(*a*) *Cohort study*—Give the eligibility criteria, and the sources and methods of selection of participants. Describe methods of follow-up<br>*Case-control study*—Give the eligibility criteria, and the sources and methods of case ascertainment and control selection. Give the rationale for the choice of cases and controls<br>*Cross-sectional study*—Give the eligibility criteria, and the sources and methods of selection of participants	3	Inclusion criteria were: (1) persistent low back pain and radiating pain or claudication in the lower extremities despite 3 months of conservative treatment; (2) radiographic evi-dence of lumbar spinal stenosis (central or foraminal) corresponding to Schizas grade C or D; and (3) availability of complete preoperative and

				postoperative imaging and clin-ical data. Exclusion criteria were: (1) a history of spine surgery at adjacent levels; (2) ra-diographic evidence of segmental instability; (3) spondylolisthesis of Meyerding grade 2 or above; (4) prior spine infection; and (5) malignant or metastatic lesions of the spine.
		(*b*) *Cohort study*—For matched studies, give matching criteria and number of exposed and unexposed<br>*Case-control study*—For matched studies, give matching criteria and the number of controls per case	—	Not applicable – this is not a matched cohort study. Groups were formed according to patients' willingness/ability to cover the incremental cost of the 3D system, not by matching (see Bias, item 9, p. 3).
Variables	7	Clearly define all outcomes, exposures, predictors, potential confounders, and effect modifiers. Give diagnostic criteria, if applicable	3	Preoperative radiographs were reviewed for spinal deformity at the operated level, which was defined as any of the following: (1) spondylolisthesis with up to 25% trans-lation on the lateral view (Meyerding grade 1); (2) scoliosis, defined as a Cobb angle greater than 10 degrees on the anteroposterior view; or (3) rotation, defined as partial disappearance of the pedicle on the anteroposterior view (Nash–Moe grade 2 or above).

Data sources/ measurement	8*	For each variable of interest, give sources of data and details of methods of assessment (measurement). Describe comparability of assessment methods if there is more than one group	5	The primary outcomes were two intraoperative time intervals, both measured from the timestamps of the archived endoscopic video recording of each procedure. Time to drilling was defined as the interval between skin incision and the start of laminotomy, and therefore reflects the time required to recognize the anatomical landmarks, achieve hemostasis, and adequately remove the overlying soft tissue. Time to flavectomy was defined as the interval between skin incision and complete removal of the ligamentum flavum, and therefore represents the total duration of the decompression. Both variables are therefore cumulative operative intervals measured from skin incision, and neither represents the duration of drilling or of flavectomy as a discrete surgical step. In addi-tion, the interval from initiation of drilling to completion of flavectomy was derived as time to flavectomy minus time to drilling. This interval isolates the deep, work-

				ing-channel portion of the procedure, but it remains a composite of several consecutive deep steps and is not a measure of flavectomy duration alone.
Bias	9	Describe any efforts to address potential sources of bias	3	Uniportal full-endoscopic unilateral laminotomy with bilateral decompression is not reimbursed by the Taiwanese National Health Insurance program, and all patients therefore self-funded the procedure at a cost of approximately NT$200,000; use of the single-lens 3D reconstructed system carried an additional charge of approximately NT$20,000. Patients whose private insurance coverage met this incremental cost were treated with the 3D reconstructed system, and all others underwent the two-dimensional procedure. All operations were performed by the same spine surgeon using an identical uniportal technique, and no patient-related, radiographic or sur-geon-preference criteria were used to select the visualization

				modality. Because cases of both types occurred throughout the entire study period rather than in separate consec-utive blocks, any effect of the surgeon's evolving experience was distributed across the two groups
Study size	10	Explain how the study size was arrived at	3	Sixty-seven consecutive patients underwent single-level uniportal full-endoscopic ULBD for lumbar degenerative disease between January 1, 2023, and December 31, 2024. Five patients did not complete 12 months of follow-up and were excluded from the final analysis, leaving 62 patients for evaluation.

Continued on next page

Quantitative variables	11	Explain how quantitative variables were handled in the analyses. If applicable, describe which groupings were chosen and why	6	To examine the effects of 3D reconstructed endoscopy use and the presence of spinal deformity on time to drilling and time to flavectomy, two-way analysis of variance (ANOVA) was performed, with endoscope type (2D vs. 3D) and deformity status (present vs. absent) as fixed factors and their interaction term included.
Statistical methods	12	(*a*) Describe all statistical methods, including those used to control for confounding	6	Descriptive statistics were used to summarize demographic and clinical data. Categorical variables were expressed as counts and proportions and compared using the chi-square test. Continuous variables were expressed as mean ± SD and compared using the Student t-test, with Welch's correction applied when variances were unequal; skewed variables were compared using the Mann–Whitney U test. Effect estimates are reported as mean differences with 95% confidence intervals, and standardized effects as Hedges' g with the small-sample correction. All analyses were performed using SPSS 26.0 (IBM, NY, USA).
		(*b*) Describe any methods used to examine subgroups and interactions	6	Two-way ANOVA was performed

			with endoscope type and deformity status as fixed factors, including their interaction term; where the interaction was significant, simple main effects were examined. Serial changes in leg VAS, back VAS, and ODI were analyzed with mixed-design repeated-measures ANOVA (time × endoscope type), with the group × time interaction as the primary test of differing recovery trajectories; significant interactions were followed by Bonferroni-adjusted post-hoc comparisons.
	(*c*) Explain how missing data were addressed	6	"All patients attended every scheduled assessment, and no outcome data were missing."
	(*d*) *Cohort study*—If applicable, explain how loss to follow-up was addressed<br>*Case-control study*—If applicable, explain how matching of cases and controls was addressed<br>*Cross-sectional study*—If applicable, describe analytical methods taking account of sampling strategy	6	Operative-time analyses included all 67 patients, including the 5 patients subsequently lost to clinical follow-up, because intraoperative data were complete for all operated patients; the VAS and ODI outcome analyses were restricted to the 62 patients with complete 12-month follow-up.
	(*e*) Describe any sensitivity analyses	—	No sensitivity analyses were

				reported.
**Results**				
Participants	13*	(a) Report numbers of individuals at each stage of study—eg numbers potentially eligible, examined for eligibility, confirmed eligible, included in the study, completing follow-up, and analysed	8	“A total of 67 patients underwent surgery, 33 with the 2D system and 34 with the 3D system, and all 67 contributed to the operative-time analyses because intraoperative records were complete for every patient (Table 1). The clinical outcome analyses were restricted to the 62 patients who completed 12-month follow-up, 31 in each group (Table 2).”
		(b) Give reasons for non-participation at each stage	3	Not specifically reported — the manuscript states only that “five patients did not complete 12 months of follow-up and were excluded from the final analysis” (§2.1); no individual reasons for non-participation are given.
		(c) Consider use of a flow diagram	—	Not applicable — no participant flow diagram was used.
Descriptive data	14*	(a) Give characteristics of study participants (eg demographic, clinical, social) and information on exposures and potential confounders	8	“The groups differed in sex distribution (2D: 24 males and 7 females; 3D: 15 males and 16 females; p = 0.018) but were comparable in age (63.6 ± 13.1 vs.

				59.4 ± 17.3 years, p = 0.290), height (158.6 ± 8.3 vs. 161.4 ± 8.7 cm, p = 0.198), and weight (55.9 ± 11.0 vs. 60.4 ± 13.8 kg, p = 0.161).” Full demographic and perioperative characteristics are given in Table 1.
		(b) Indicate number of participants with missing data for each variable of interest	6, 8	“All patients attended every scheduled assessment, and no outcome data were missing” (p. 6). Demographic comparisons (age, sex, height, weight, hospital stay) “could be computed only for the 62 patients with complete records” (p. 8); the remaining 5 patients contributed complete intraoperative data only.
		(c) *Cohort study*—Summarise follow-up time (eg, average and total amount)	6	Outcome questionnaires were “collected on the day before surgery and at 1, 3, 6, and 12 months postoperatively within a window of ±2 weeks for each time point.” Clinical follow-up was therefore fixed at 12 months for all 62 analyzed patients.
Outcome data	15*	*Cohort study*—Report numbers of outcome events or summary measures over time	8–9	“All three intraoperative intervals favored the 3D group. Time to drilling was shorter with the 3D

				system (6.1 ± 2.6 vs. 4.4 ± 1.2 minutes, p = 0.001), as was time to flavectomy (61.4 ± 8.0 vs. 41.8 ± 7.3 minutes, p < 0.001), representing reductions of 29% and 32%, respectively.” Full outcome data are given in Tables 1 and 2.
		Case-control study—Report numbers in each exposure category, or summary measures of exposure		
		Cross-sectional study—Report numbers of outcome events or summary measures		
Main results	16	(*a*) Give unadjusted estimates and, if applicable, confounder-adjusted estimates and their precision (eg, 95% confidence interval). Make clear which confounders were adjusted for and why they were included	9–11	The interval from initiation of drilling to completion of flavectomy was 17.87 minutes shorter with 3D (95% CI 14.1–21.6; Hedges' g 2.29, 95% CI 1.67–2.91; p < 0.001), stratified as 14.63 minutes shorter in the absence of deformity and 22.28 minutes shorter in its presence (p. 9). At 12 months, ODI was 6.3 percentage points lower with 3D (adjusted difference, 95% CI 3.0–9.5; p < 0.001), while back and leg VAS did not differ significantly between groups (adjusted differences with 95% CI; pp. 10–11).
		(*b*) Report category boundaries when continuous variables were categorized	3	Spinal deformity, the categorized effect modifier examined in the main analyses, was defined as any of: spondylolisthesis with up to

			25% translation (Meyerding grade 1); scoliosis with a Cobb angle >10°; or rotation (Nash–Moe grade ≥2) — see Variables, item 7.
	(*c*) If relevant, consider translating estimates of relative risk into absolute risk for a meaningful time period	—	Not applicable — effects are reported as mean differences and standardized effect sizes (Hedges' g) for continuous outcomes, not as relative risks.

Continued on next page

Other analyses	17	Report other analyses done—eg analyses of subgroups and interactions, and sensitivity analyses	9	"For time to drilling, the effect of the 3D system did not depend on the presence of deformity (interaction $F(1,63) = 1.98$, $p = 0.165$)... For time to flavectomy, the effect of the 3D system did depend on deformity status (interaction $F(1,63) = 6.79$, $p = 0.011$), indicating that the 3D system largely offset the additional difficulty of deformed anatomy."
**Discussion**				
Key results	18	Summarise key results with reference to study objectives	12	"Three findings emerged. First, the 3D system shortened both intraoperative intervals measured, most markedly for flavectomy and in patients with segmental deformity. Second, pain relief was equivalent. Third, functional recovery was comparable overall, although the ODI trajectories diverged in favor of the 3D group."
Limitations	19	Discuss limitations of the study, taking into account sources of potential bias or imprecision. Discuss both direction and magnitude of any potential bias	14	"This study has several limitations. First, allocation was not randomized but determined by insurance coverage... Second, the sex distribution differed significantly between the two groups. Third, no a priori sample

				size calculation was performed... Fourth, all procedures were performed by a single surgeon over a two-year period... Sixth, follow-up was both short and incomplete... Seventh, time to drilling and time to flavectomy are cumulative intervals... Eighth, surgeon-side tolerability of the stereoscopic display was not assessed systematically... Finally, no cost-effectiveness analysis was performed.”
Interpretation	20	Give a cautious overall interpretation of results considering objectives, limitations, multiplicity of analyses, results from similar studies, and other relevant evidence	13, 16	“This difference is also smaller than the accepted MCID for the ODI in this population, so it should be regarded as hypothesis-generating rather than as evidence of a clinically meaningful functional benefit” (p. 13). “The practical implication is narrower than a general claim of superiority... they should not, however, expect a patient-reported outcome benefit, because the functional advantage observed fell below the MCID and arose from an interaction analysis” (Conclusions, p. 16).
Generalisability	21	Discuss the generalisability (external validity) of the study results	14, 16	“All procedures were performed by a single surgeon over a two-year

				period. Although 2D and 3D cases were distributed across the entire study period, a learning effect over time cannot be fully excluded” (p. 14). “Surgeons already proficient in 2D uniportal ULBD can expect the largest return where segmental deformity distorts the interlaminar anatomy...” (Conclusions, p. 16).
**Other information**				
Funding	22	Give the source of funding and the role of the funders for the present study and, if applicable, for the original study on which the present article is based	17	“Funding: This research received no external funding. The APC was funded by Y.-H.C.”

*Give information separately for cases and controls in case-control studies and, if applicable, for exposed and unexposed groups in cohort and cross-sectional studies.

Note: An Explanation and Elaboration article discusses each checklist item and gives methodological background and published examples of transparent reporting. The STROBE checklist is best used in conjunction with this article (freely available on the Web sites of PLoS Medicine at http://www.plosmedicine.org/, Annals of Internal Medicine at http://www.annals.org/, and Epidemiology at http://www.epidem.com/). Information on the STROBE Initiative is available at www.strobe-statement.org.
